# Considerations on the First Traces of the Osseous System, and on the Development of the Vertebral Column in Animals

**Published:** 1820-06

**Authors:** C. A. S. Schultze


FOR THE LONDON MEDICAL AND PHYSICAL JOURNAL.

### *Considerations on the First Traces of the Osseous System, and on the Development of the Vertebral Column in Animals.* By Dr. C. A. S. Schultze.

THE term *bone*, or *osseous structure*, taken in its most extended acceptation, has been employed, since the origin of anatomy, to designate all the hard, and most frequently white, parts of the animal economy which are manifestly of an organic tissue, and in the composition of which calcareous matter predominates. It is clear that the expression *osseous system*, designating a whole produced by the union of those parts, conveys a different idea; but it is also certain, that this definition is not sufficient, when we wish to indicate the first vestiges of the osseous system at a time when the colour, the form, and the composition, of it are not the same, and especially when its connexions and relations do not yet exist: in this case, the functions of each part can alone throw any light on its nature. It is from this consideration having been neglected, and from different ideas having been attached to the terms *bone* and *osseous system*, that several arrangements and systems have been established which are by no means conformable to nature.

The section of the animal kingdom which may be considered as nearly the best distinguished, is marked by the first traces of the osseous system, though this limit has recently lost a little of the precision it was formerly supposed to possess. However, of all the systems, the osseous mechanism is that which appears the latest: it is only seen in the more perfectly organized animals, as a protective envelopment of the brain and spinal marrow. It is thus that this system, which is almost inorganic, and the whole of which creates the idea less of an organism than that of a pure and simple mechanism, becomes one of the conditions of the most remarkable of all the vital phenomena. The phenomena of sensibility, exalted to the point of the production of thought, cannot take place but by the medium of the osseous apparatus.

It is almost generally admitted, that the principal functions of the osseous system, is the regulation of the form of the body, and the facilitation of its movements; but it is only necessary to turn our eyes on insects, to be assured that an exterior skeleton would fulfil these two functions in a much better manner. Insects are the animals in which voluntary movement and permanence of the form of the body have been carried to the highest degree. Now nature would certainly not have renounced a disposition so favourable to motion, and which furnishes such solid points of support to the rest of the body, if it had not some other end in the creation of an interior skeleton. It is only necessary to study the latter with attention, especially in its least complicated forms, to become convinced that its principal destination is to form a firm envelopment for the central part of the nervous and vascular systems; to render, thus, the actions of these parts more free, and especially to establish a boundary, a line of demarcation, between them and the muscles.* If it also serve as a passive organ for movement, this is only an inferior function; for, should all the bones become softened in an animal of one of the superior classes, it would not be the impossibility of motion, but the compression of the organs essential to life, that would occasion death. It may be further stated, in support of these views,

1°. That the spinal marrow and vertebral column at all times exist together, even when only the slightest vestiges of the osseous system can yet be found.

2°. That the osseous and nervous systems have between them numerous intimate relations, both physiological and pathological.

3°. That the more the exterior hard envelopment penetrates the interior of the body, and approaches towards the nervous system, the more also are the phenomena of sensibility developed, and *vice versa.*†

* This opinion had been previously advanced by Sir Everard Home, in his Lectures on Comparative Anatomy; and it has been adopted by Geoffroy Sainte-Hilaire and Blainville.

† It is thus that the brain performs so secondary a part in the tortoise, and that, in this respect, as well as in that of their shell, the chelonians so nearly approach the class of insects.

4°. That the organs possess more or less importance, according as they are more or less protected from nervous influence by bones.

According to the foregoing principles, only those parts which serve to envelop the central portions of the nervous and vascular systems, or on the surface of which the muscles of the organs of motion are attached, can be considered as corresponding to the osseous system, properly speaking. Thus, the hard parts of all the zoophytes, particularly those of the asteria and the ophima, cannot relate to it, although they may be moved by muscles situate between them: these are only shells, which, still united in the ursina, entirely envelop the animal. So the different parts of the cuirass of insects and the crustaceæ do not present the essential character above mentioned, although we must acknowledge that they have some analogy with the parts of the skeleton of vertebral animals, to which they even correspond to a certain point in their situation and connexions.

It is otherwise with respect to the development of the teeth, and the apparatus which supports them. These bones are found in a great number of invertebral animals, often indeed very strongly developed;* and, in their quality of parts constituent of the alimentary canal, to which they are attached, they correspond to the same parts in vertebral animals, in which it is only more common to see them border the opening of the mouth, and situate either in front or below the cranium. Even n the most simple of these animals, for example, in the lamprey, they are still separated from the cranium, that is to say, from the vertebral system, with which they become united and confounded only by successive gradations in different animals. This union afterwards subsists in all vertebral animals up to man; and it is the expression of the relative proportion which exists, on the one part between the brain and cranium, and on the other between the intestinal canal and the jaws. I should deviate from my plan, if I were to dwell longer on the consideration of these parts. I return, then, to the osseous system, taken in the most restricted sense of the term, to the system which might be termed *vertebral*, because of the analogy of all its parts with the entire vertebræ, or parts of the vertebræ.

Analogy shows us that we may apply the history of the development of an isolated individual to that of the whole of the animal chain. Now, as the production of the cartilaginous tissue always precedes that of the osseous tissue in all the ver-

---

* The teeth present infinite varieties, with respect to their form and situation, in invertebral animals, but very few species of which are entirely deprived of these bones and of jaws. Besides the organs of manducation apparent externally, the teeth, or the osseous plates which line the stomach of several of them, and which, at least the latter, are perhaps the rudiments of the branchial arches of fishes, should be here included.

tebræ, so also does it appear to precede it, as a constant grade of organization, to the extreme limit of this great series.

The cephalopodes amongst the molluscæ, rendered so remarkable by the complete development of the nervous system and several organs of the senses, are the animals which appear to present the first rudiments of some parts of the skeleton. They are thus disposed in the sepia:

The bone of the sepia that Spix regards as a rudiment of the vertebral column, is evidently not a part of that structure: it is produced by the mantle of the animal, and, consequently, is analogous to the concha of the other molluscæ, as is proved by Cuvier. As the sepia appertains in one respect to the molluscæ, and approaches in another to the more perfect animals, so the cephalic cartilage, irregular in its figure, presents in it a very remarkable union of the form of the cranium of vertebral animals with that which appertains to the brain of invertebral animals, which surrounds the œsophagus like a ring. The cranium has consequently the form of a ring, traversed by the œsophagus, the salivary ducts, &c. It is cartilaginous outwardly, and purely membranous inwardly. Behind its lower part, which surrounds the medullary ring, it is united to a cavity formed of thick parietes, and which constitutes the organ of hearing. On the two sides, it is enlarged to form the posterior and inferior parietes of the orbits, which are incompletely closed in front by two thin and oblong cartilaginous plates. These two plates, united in the middle, spring from the anterior and inferior part of the annular cartilage. Meckel thinks that they correspond to the superior maxillary bones of vertebral animals. Immediately in front of the whole of this cartilaginous mass, at the basis of the foot which envelops the beak, we perceive, on the side of the belly, a flat and almost triangular cartilaginous piece, the base of which is directed towards the feet, and the summit towards the ring, to which it is attached only in a loose manner by muscular fibres.

Spix regards this plate as the rudiment of the os hyoides; but Meckel, perhaps with more propriety, considers that it corresponds to the lateral and posterior parts of the lower jaw, at least if the inferior half of the beak be regarded as the middle portion of this jaw, and its superior half as the intermaxillary bone. Behind the cartilaginous ring which covers the brain, we perceive, on the back, a small cartilaginous plate, pointed in front, notched behind, and traversed by a groove throughout the whole extent of its middle part. On this plate there is another, thinner, of the same figure, and having a protuberance on one side corresponding to the groove of the former. This plate covers the œsophagus, the inferior salivary glands, and some muscles which run to the cephalic cartilage, but of which several fasciæ

are attached to its inferior surface. The other is situate under the lower extremity of the bone, in its capsule: posteriorly, it is lost in this membranous envelopment; on the sides, it is confounded with its cartilaginous margins, without even being separated by cellular tissue.

The use of these two plates is, evidently, to protect the head and the soft parts it encloses from the weight of the whole body, and especially of that of the bone, and to prevent friction on it. According to Meckel, the first represents the annular portion of the vertebral column: he raises this notion not only on the situation of the body behind the cephalic cartilage, and on the form it assumes in the calmar, but also on this fact,—that the annular portion of the vertebræ is much more developed than the body in cylostomic fishes, ranked immediately above the cephalopodes. But I think that this plate appertains to the integuments or to the shell, and my arguments are: 1°, the absence of the spinal marrow; 2°, the situation of the piece immediately over the cutaneous muscles, several of which are even attached to its inferior part, for the purpose of retaining it in its place; 3°, its absence in the *octopus*, where the shortness and softness of the body would render it really useless. Perhaps it corresponds to the dorsal shell of lobsters, which the cephalopodes approach in many respects.

In the calmar, only a very thick cartilage is found corresponding to the first of these two plates, which has a prominence in its middle receiving the anterior extremity of the horny shell, which is hollowed into a sort of channel.

The cartilages which support the fins bring the cephalopodes still nearer to vertebral animals, according to Meckel. These are two long and thin cartilaginous plates, the internal surface of which, concave and polished, is fixed on the side of the mantle; below, by the thin and external lamina of the cartilage itself; and above, by the muscles which spring from the mantle. Its external and convex surface is divided into two portions by a longitudinal crest: one, inferior, is smooth and polished; the other, superior, serves for the insertion of muscles. In the sepia, they occupy, like the fins, the whole length of the mantle, and, becoming larger and thicker posteriorly, they terminate very near to each other. In the calmar, their length does not exceed that of the base of the fins.

Besides these parts, we also find, at each of the external angles of the base of the tunnel, a cartilage, hollowed out by a cavity which answers to a fleshy protuberance at the internal surface of the mantle. Cuvier stated that the object of this disposition was to render the mantle more firm and solid. It corresponds, then, on the side of the belly, to the cartilaginous apparatus situate at the back, and cannot be any more assimilated to any of the parts of the osseous system of vertebral animals, than the cartilaginous rings situate in the suckers of the arms.

However different this construction may be from that of fish, exteriorly as well as interiorly, there still exist intermediate parts which diminish to a certain extent the gap in the chain.

The genus gastobranchia, although nearer to red-blooded worms by its form and organs of respiration,* presents, however, at the margin of the tunnel leading to the mouth, eight free filaments, which surround this opening, and which represent the feet of the cephalopodes rather than organs of touch, and they also fulfil an analogous function. They have merely experienced a diminution in length proportionate to the increase that the tail, become the sole organ of movement, and charged also with part of the functions of the feet, has received towards the other extremity of the body. The suckers are replaced by the entire mouth, become a vast sucker, at the bottom of which are seen the horny jaws, with serrated margins.† But, it is here that we find the first rudiment of a true vertebral column containing a spinal marrow.

The spine, or vertebral column, consists, as Oken had already remarked,‡ in an osseous column bordering the sides of the body, divided into superior or dorsal, and inferior or ventral, each having a canal, running its whole length, formed by arches which emanate from it. This column is, most frequently, the result of an assemblage of isolated pieces, of a cylindrical form, and attached to each other. The superior canal contains the spinal marrow; the organs of respiration and digestion are lodged in the second or ventral canal. The latter, in respect to the viscera it contains, has arches much larger and more detached from each other than the former. It is rare that these arches unite directly together to produce complete rings: this, however, takes place in some lizards and serpents; they are either entirely free in front, or they are united by the medium of a series of bones, disposed from before backwards, and which is called the sternum. However, in the latter series in the chain of vertebral animals, the inferior canal is contracted towards the posterior part of the vertebral column, or it only contains vascular trunks; so that it perfectly resembles the superior canal. This symmetrical disposition disappears in proportion as we rise in the scale; for it is one of the principal

* See the Memoir of Sir Everard Home on the structure of the respiratory organs in animals which appear to be intermediate to fish and worms, in the Philosophical Transactions for 1815.

† The barbs which are so often found round the mouth of fishes, mark this point of resemblance with the cephalopodes.

‡ *Lehrbuch der Naturphilosophie*, tom. iii. p. 65.

characteristics of the perfection of the osseous system, that the arches of the inferior canal, which are in general the least constant, gradually close together from distance to distance, and disappear in the intervals; whilst, on the contrary, those of the superior canal, which most frequently correspond in number to the bodies of the vertebræ, extend almost uniformly to the extremity of the vertebral column, or at least arrive very near to it. These are called vertebral arches; the others bear the name of ribs or transverse apophyses, when they surround the respiratory and digestive organs; and that of inferior vertebral arches, when they serve only to protect the vascular trunks. Very commonly these last inferior arches, and all the superior ones, support, at the point where the branches of opposite sides join each other, spinous apophyses, which serve for the insertion of muscles. In general it may be stated, it is the viscera which determine the general and fundamental form of the two rings, but it is the different species of movement which occasion almost all the changes of form, even in the vertebral column.

In the points where the inferior arches concentrate, the limbs are formed, which extend in the fluid in the midst of which the animal lives, and which vary in form according to the nature of this fluid. It is according to these points of concentration that the vertebral column is divided into several portions, to which the names now generally received, being taken from the state of things in man, only apply when the limbs are completely developed, and when there are, in succession, an absence and considerable development of the arches. Thus, we distinguish *cervical* vertebræ, deprived of ribs; *dorsal* and *pectoral*, which possess them; *lumbar*, also without ribs; *sacral* or *pelvic*, which support the bones of the pelvis, analogous to the ribs; and, lastly, the *coccygean* or *caudal*, which do not support, it is true, any ribs, but which, in many animals, are furnished with an inferior canal containing an arterial trunk. This division is applicable in its full extent only to reptiles, birds, and the mammalia provided with the bony elements, at least, of the limbs; since, in particular, the three last sections of the vertebral column recognize no other limits than those of the connexions which the last but one of them has with the pelvic extremities.

We can distinguish, then, in all fishes, as well as in reptiles and the mammalia deprived of posterior extremities, only *dorsal*, *caudal*, and often one or more *cervical*, vertebræ: the last are situate nearest to the head, and do not support any ribs. The caudal are furnished with an inferior vertebral canal for the vascular trunk. Between these and the preceding ones, we find the dorsal, which may sometimes be distinguished into those which support ribs, and those which are devoid of them. It is difficult, and even almost impossible, to establish general rules in regard to the relations existing between the different divisions of the vertebral column in different animals; for, at the bottom of the scale, the skeleton presents more inconstancy and variation in its form, its texture, its structure, and its functions, than any other system does at the grades where it shows itself for the first time. It is sometimes one, and sometimes the other, of those divisions which is developed superiorly to the rest, and this development is sometimes a step further towards a more perfect form; whilst, at others, it is only a modification produced by the mode of life, or even a retrograde step, and a return towards the more rude and imperfect forms. However, the following corollaries are susceptible of a nearly general application.

1°. The caudal vertebræ are the least developed of the whole series.

2°. The number and length of all the vertebræ are ordinarily in an inverse ratio to the development of the limbs.*

3°. The number and length of the cervical vertebræ are in a direct ratio to the development of the fore limbs; whilst the number, the length, and the height, of the caudal vertebræ, are in an inverse ratio to the development of the two pair of limbs, inferior as well as posterior.

4°. The number of the lumbar and pelvic vertebræ is proportionate to the development of the posterior extremities; that of the pectoral or dorsal vertebræ, properly speaking, is the same in respect to the development of the thoracic members.

5°. The number of vertebræ continues the same in warm-blooded animals during the whole of their life, that is to say, from their birth to their death: it is not so with all cold-blooded animals; the number of the caudal vertebræ increasing regularly for the whole duration of life in some instances.

